# SOCIAL DETERMINANTS OF HEALTH AND DEMENTIA: PUBLIC HEALTH PERSPECTIVES

**DOI:** 10.1093/geroni/igad104.0270

**Published:** 2023-12-21

**Authors:** Joseph Gaugler, Lisa McGuire, Fayron Epps

## Abstract

The U.S. Department of Health and Human Services and its Office of Disease Prevention and Health Promotion define social determinants of health (SDOHs) as “the conditions in the environments where people are born, live, learn, work, play, worship, and age that affect a wide range of health, functioning, and quality-of-life outcomes and risks.” Featuring presenters from the three national Building our Largest Dementia Infrastructure (BOLD) Public Health Centers of Excellence as well as other Centers of Disease Control and Prevention Healthy Brain Initiative programs, this symposium will examine how dementia influences, and is influenced by, SDOHs. Specifically, issues that require critical public health action, including prevention, early detection, and family caregiving will be examined within a SDOH framework. Transcending these concerns, this symposium will also feature a presentation that considers the complex associations between health equity, SDOHs, and dementia. We anticipate that this symposium and its presentations will facilitate and advance gerontology’s understanding of dementia as a public health imperative, and as importantly, begin to identify how public health at the local, tribal, state, and federal levels can mobilize to address the challenges and opportunities of dementia.

